# Supplementary material for: Impact of Education on Metabolic Dysfunction-Associated Steatotic Liver Disease (MASLD): A Southern Italy Cohort-Based Study
Source: J Clin Med. 2025 Mar 13;14(6):1950. doi: 10.3390/jcm14061950 (PMC11943323; doi:10.3390/jcm14061950)
Supplement: Supplementary file 1 [file jcm-14-01950-s001.zip › jcm-3446445-supplementary.pdf]

Journal of Clinical Medicine

Supplementary Material

Table S1. Mediation analysis of socio-demographic parameters on MASLD and education as mediator.

Effects	β	se (β)	95% C.I.	p
Indirect				
Gender → Education → MASLD	0.005	0.004	-0.003 to 0.013	0.257
Age → Education → MASLD	0.067	0.010	0.050 to 0.089	<0.001
Smoking → Education → MASLD	0.006	0.006	-0.003 to 0.020	0.158
Job → Education → MASLD	0.051	0.003	0.012 to 0.023	<0.001
Marital Status → Education → MASLD	0.014	0.004	0.004 to 0.021	0.003
Kcal → Education → MASLD	-0.001	2.84e-6	-6.31e-6 to 4.81e-6	0.792
Income → Education → MASLD	-0.035	0.002	-0.017 to -0.009	<0.001
Component				
Gender → Education	-0.024	0.038	-0.119 to 0.031	0.252
Education → MASLD	-0.204	0.014	-0.138 to -0.083	<0.001
Age → Education	-0.327	0.041	-0.713 to -0.0551	<0.001
Smoking → Education	-0.029	0.054	-0.184 to 0.028	0.151
Job → Education	-0.250	0.013	-0.185 to -0.131	<0.001
Marital Status → Education	-0.07	0.035	-0.185 to -0.05	0.001
Kcal → Education	0.005	2.57e-5	-4.36e-5 to 0.005	0.792
Income → Education	0.173	0.014	0.090 to 0.146	<0.001
Direct				
Gender → MASLD	-0.144	0.023	-0.189 to -0.100	<0.001

(continue)

Age → MASLD	0.199	0.026	-0.156 to 0.259	<0.001
Smoking → MASLD	0.006	0.032	-0.054 to 0.072	0.774
Job → MASLD	-0.002	0.008	-0.017 to 0,016	0.945
Marital Status → MASLD	0.003	0.021	-0.039 to 0.044	0.902
Kcal → MASLD	-0.024	1.53e-5	-4.62e-5 to 1.36e-5	0.286
Income → MASLD	0.020	0.008	-0.009 to 0.024	0.375
Total				
Gender → MASLD	-0.139	0.023	-0.185 to -0.094	<0.001
Age → MASLD	0.265	0.025	0.228 to 0.326	<0.001
Smoking → MASLD	0.012	0.033	-0.046 to 0.082	0.586
Job → MASLD	0.049	0.008	7.95e-4 to 0.033	0.040
Marital Status → MASLD	0.017	0.021	-0.026 to 0.057	0.472
Kcal → MASLD	-0.025	1.55e-5	-4.74e-5 to 1.34e-5	0.273
Income → MASLD	-0.015	0.008	-0.022 to 0.011	0.513

Abbreviations: β, Coefficient; se (β), Standard Error of β; 95% C.I., Confidence Intervals at 95%.
